# Associations between glucocorticoids and habitat selection reflect daily and seasonal energy requirements

**DOI:** 10.1186/s40462-024-00475-9

**Published:** 2024-04-22

**Authors:** Levi Newediuk, Gabriela F. Mastromonaco, Eric Vander Wal

**Affiliations:** 1 Department of Biology, Memorial University, St. John’s Newfoundland, A1B 3X9 Canada; 2 Reproductive Physiology, Toronto Zoo, Toronto, ON M1B 5K7 Canada; 3 Current address: Biological Sciences Department, University of Manitoba, Winnipeg Manitoba, R3T 2N2 Canada

**Keywords:** Stress, Hormones, Physiology, Fitness, Integrated step-selection analysis, State-dependent habitat selection

## Abstract

**Background:**

Glucocorticoids are often associated with stressful environments, but they are also thought to drive the best strategies to improve fitness in stressful environments. Glucocorticoids improve fitness in part by regulating foraging behaviours in response to daily and seasonal energy requirements. However, many studies demonstrating relationships between foraging behaviour and glucocorticoids are experimental, and few observational studies conducted under natural conditions have tested whether changing glucocorticoid levels are related to daily and seasonal changes in energy requirements.

**Methods:**

We integrated glucocorticoids into habitat selection models to test for relationships between foraging behaviour and glucocorticoid levels in elk (*Cervus canadensis*) as their daily and seasonal energy requirements changed. Using integrated step selection analysis, we tested whether elevated glucocorticoid levels were related to foraging habitat selection on a daily scale and whether that relationship became stronger during lactation, one of the greatest seasonal periods of energy requirement for female mammals.

**Results:**

We found stronger selection of foraging habitat by female elk with elevated glucocorticoids (e^ß^ = 1.44 95% CI 1.01, 2.04). We found no difference in overall glucocorticoid levels after calving, nor a significant change in the relationship between glucocorticoids and foraging habitat selection at the time of calving. However, we found a gradual increase in the relationship between glucocorticoids and habitat selection by female elk as their calves grew over the next few months (e^ß^ = 1.01, 95% CI 1.00, 1.02), suggesting a potentially stronger physiological effect of glucocorticoids for elk with increasing energy requirements.

**Conclusions:**

We suggest glucocorticoid-integrated habitat selection models demonstrate the role of glucocorticoids in regulating foraging responses to daily and seasonal energy requirements. Ultimately, this integration will help elucidate the implications of elevated glucocorticoids under natural conditions.

**Supplementary Information:**

The online version contains supplementary material available at 10.1186/s40462-024-00475-9.

## Introduction

A key question in animal ecophysiology is whether glucocorticoids, the so-called “stress” hormones, drive behaviours that increase fitness or indicate exposure to stressors that might compromise fitness. Negative relationships often emerge between glucocorticoids and fitness because glucocorticoid production is a common response among vertebrates to dealing with stressful environments [1]. Stress, however, is something all organisms have evolved to deal with, suggesting the conserved production of glucocorticoids among all vertebrates is adaptive [2, 3]. Consistent with this adaptive theory of glucocorticoid production, many other competing hypotheses predict that glucocorticoid production instead increases fitness by supporting the behaviours individuals use to continue to survive and reproduce in stressful environments [4]. The expectation is that glucocorticoids ultimately drive the best strategies to improve long-term fitness [1, 5].

One of the ways glucocorticoids are thought to support fitness is by driving foraging behaviours to meet changing daily and seasonal energy requirements. Glucocorticoids bind to receptor sites that promote muscle catabolism, limit the secretion of appetite-suppressing hormones, and increase blood glucose, all of which increase the sensation of hunger [6]. The result can be an increase in feeding behaviour [7]. Because of its effects on feeding, glucocorticoid production follows predictable circadian spikes corresponding to peak daily energy requirements [8, 9]. Rearing offspring typically increases daily energy requirements, and consequently, many species either produce their highest glucocorticoid levels of the year during their reproductive seasons [10] or alter other physiological pathways to promote glucocorticoid binding. For example, the production of corticosteroid-binding globulins (CBGs) is sometimes reduced during reproduction [11]. CBGs compete with receptors to bind glucocorticoids in the bloodstream; reducing CBGs means more glucocorticoids are “free” to bind receptors controlling behavioural changes, even without any change in glucocorticoid concentration [12].

The first step in establishing whether glucocorticoids reflect stressful environments or energy requirements is to test for a relationship between glucocorticoids and daily and seasonal foraging behaviour. Habitat selection models are a well-established tool for assessing which variables influence behaviours related to space use. Traditionally, habitat selection models measure how characteristics of habitats influence which are selected or avoided [13]. However, recent interest in behavioural differences between individual animals has inspired innovative new models quantifying the effects of dynamic social environments [14], behavioural states [15], and disease [16] on habitat selection. Another natural extension of habitat selection models would be to quantify relationships between glucocorticoids and the selection of habitats used for foraging as energy requirements change.

We used habitat selection models to test the relationship between glucocorticoids and foraging habitat selection of female elk (*Cervus canadensis*) responding to daily and seasonal changes in energy requirements. We hypothesized that variation in glucocorticoids sampled under natural conditions reflects individual daily and seasonal differences in energy requirements. In our study population, the densest source of forage for elk is cropland, with less forage available from habitats like forests and shrubland that make up the surrounding landscape. Elk are known to both prefer to forage in cropland when available [17] and gain more weight by foraging in cropland relative to natural habitats [18]. Our observations and those of others in our study population (e.g., Hinton et al [19]), and nearby populations [20] suggest elk make daily movements between forests and shrubland habitats where they rest and cropland where they forage. Thus, we first predicted that elevated daily glucocorticoid levels in elk would be associated with subsequently stronger selection for cropland habitat, i.e., high-quality foraging habitat, relative to forests and shrubland.

We also predicted elevated glucocorticoid levels and changes in habitat selection would be associated with seasonal changes in the energy requirements of female elk. Like many female mammals, elk face their largest seasonal energy requirements while lactating [21]; female elk must maintain a minimum over-winter body condition to calve in the spring, but lactation and calf growth depend almost exclusively on summer foraging [22]. The energy requirements of female elk consequently increase several-fold between the last day of gestation and the first day of lactation [23]. To support these energy requirements, we predicted mean glucocorticoid levels would increase immediately after calving.

Glucocorticoids could also support larger energy requirements after parturition without mean levels necessarily increasing. In other species, annual glucocorticoid levels remain consistent, but CBG concentrations decline seasonally which renders more receptor sites available for binding (e.g., Love et al [11]). Changes in receptor availability should modify behavioural decisions like habitat selection. Without being able to measure CBGs directly, we instead predicted that mean glucocorticoid levels would remain the same after calving, but peak glucocorticoid levels after calving would be associated with stronger selection for cropland. We also predicted the relationship might become stronger with time post-calving to match the energetic requirements of the growing calf.

## Materials and methods

### Elk captures

We used global positioning system (GPS) locations of adult female elk to characterize habitat selection and identify calving sites. In February 2019, a crew captured 13 adult female elk in southeast Manitoba, Canada (49.134, -96.557) from a population of approximately 150 individuals. They extracted a blood sample from all individuals and fitted each with a GPS collar (Vertex Plus 830 g, VECTRONIC Aerospace GmbH, Berlin, Germany) that collected locations every 30 min during the calving season (May through July). Each day from May–August 2019 and 2020, we visually monitored GPS movement patterns for signs of the collared elk having given birth. Elk calves hide for 4–5 days following parturition until they are mobile enough to escape predators [24]. This limited mobility causes elk mothers to reduce their own movement rates to remain close to their calves [20]. We inspected suspected calving sites based on slow movements of female elk, and when we located calves, we fit them with a very high frequency (VHF) radio collar (V6C 83 g, Lotek, Newmarket, Ontario, Canada) for monitoring survival. Both adult female and calf capture procedures were in accordance with approved animal care protocols (Memorial University of Newfoundland animal use protocol #19-01-EV).

### Estimating calving dates

Using a machine-learning approach, we modelled the frequency of return visits to observed calving sites to estimate the locations and dates of unobserved calving events, i.e., those we could not find by visually inspecting the data [25]. We monitored the 13 elk over both the 2019 and 2020 calving seasons, meaning we expected to observe up to 26 calving events. We processed all data and performed all statistical analyses using R v4.3.1 [26]. We used the *recurse* package v1.3.0 [27] to calculate the number of return visits by each elk within a buffer surrounding its location points between May 15 and July 20 in both 2019 and 2020. Unlike some other ungulate species, elk calves select new hiding spots away from the calving site shortly after parturition [28], meaning mothers might make return visits to different locations. To account for variation in return location, we used a 300 m radius buffer [29] to calculate recursive movements to the calf rather than the 100 m radius buffer suggested in Marchand et al. [25].

We used elk movements surrounding 11 observed calving events as training data to predict an additional 15 potential unobserved events. We defined calving events as the time between the observed calving date up to 5 d following to account for the most intensive hiding phase. After down sampling the training data to balance the number of points within and outside the 5-d calving event, we used a random forest classifier to predict the probability of each training data point belonging to the calving event. We averaged the probability of calving for each point falling within observed calving events and used this as a threshold for detecting unobserved calving events in the testing data. Specifically, we located where average probabilities exceeded the known calving threshold within a 5-d rolling window in the testing data. After repeating this process 100 times, we selected the 5-d window of points with the highest probability of belonging to each calving event. We set the estimated calving date as the first date within the calving event window.

### Hormone sampling

We collected 154 fecal pellet samples to monitor glucocorticoid levels of the 13 collared elk from May–August 2019 and 2020. We identified clusters of location data indicative of bedding during the calving period, a time when female elk typically isolate for several days before and after parturition before they join small nursery herds with a few other elk and their calves [24]. Their relative isolation minimized the possibility that we collected a sample from an uncollared elk, and in no instance did we find evidence of more than three other elk having recently been within 20 m of a cluster [30]. After confirming bedding by visiting the locations within 24 h of the individual being present in the area, we collected any visible fecal material.

Fecal glucocorticoid metabolites (FGMs) measured in fecal pellets are used as a proxy for circulating glucocorticoids. FGMs are the product of circulating glucocorticoids metabolized over a period of hours to days [31], making FGMs an integrated measure of the peaks and troughs of circulating glucocorticoids hours to days before defecation [32]. However, FGM recovery from fecal pellets is also influenced by environmental factors acting on fecal samples after defecation [33]. For example, FGMs degrade when samples become wet in the field and when they are stored improperly after collection [34]. To minimize environmental error, we avoided sampling after rain, collected samples within 24 h of suspected defecation, and froze samples at -20° C as soon as possible (< 8 h) after collection [35], keeping them frozen until we quantified hormones.

We extracted FGMs from fecal samples following the procedure described by Morden et al. [36]. In brief, we oven-dried and homogenized fecal samples then extracted FGMs with 80% methanol: water (v: v) at a ratio of 0.04 g/ml rotating overnight. We measured FGMs using a cortisol enzyme immunoassay previously described by Majchrzak et al. [37, 38]. Cortisol antibody and cortisol horseradish peroxidase dilutions were 1:10,250 and 1: 33,400, respectively. The cortisol antibody (R4866) cross-reactivities were 100% to cortisol and < 10% with other metabolites.

### Identifying individual samples

Integrating glucocorticoids into habitat selection models requires new sampling approaches. Outside of experimental settings, glucocorticoids are often either sampled once from individuals during capture or continuously from hair, feces, and other materials left behind by animals in their environments. However, one-time samples from individuals lack the temporal resolution to track daily and seasonal rhythms in glucocorticoids [10] and samples collected non-invasively from the environment risk conflating common among-individual variation in glucocorticoids with meaningful variation [39]. We overcame these issues by training a machine-learning model to assign non-invasively collected samples to DNA-identified individuals from specific points in time using their biotelemetry data [30].

We identified individuals by comparing DNA extracted from fecal samples to that from whole blood samples taken from individuals at the time of capture. Like FGM concentrations fecal DNA is susceptible to degradation from inclement weather and storage conditions. In our case, only approximately 20% of extractions were successful. For those samples we could not identify using DNA (122 of 154 samples), we used supervised machine learning to assign suspected individuals to samples based on movement patterns and level of elk activity in the vicinity of the sample. The training model identified whether samples belonged to the suspected individual with 77% accuracy [30]; see also for further details on DNA extraction and machine learning models). We used this accuracy as a threshold for correct identification, predicting the accurate identification of testing samples over 500 iterations. We assumed samples belonged to the suspected individual when the mean predicted accuracy of testing samples exceeded the threshold accuracy.

When the mean predicted accuracy was less than the threshold, we tested whether samples could have belonged to a different collared individual in the same area around the time of defecation. We identified candidate individuals as those with any location points within 20 m of the sample up to 2 d before the time of sample collection. We repeated the same machine learning procedure for these new individuals, replacing the original individual that did not meet the threshold for correct identification with the new suspected individual. As before, we assumed samples belonged to the new individual if the predicted accuracy across 500 iterations exceeded the threshold accuracy.

### Statistical analysis

We tested for differences in glucocorticoid levels sampled before and after calving events using a Bayesian generalized linear model. Our model included two categorical variables: period before and after the calving event according to when samples were deposited by the elk relative to the known or assigned calving date, and year to account for possible between-year differences. We used FGMs as the response variable. We also included random intercepts for individuals to account for individual differences in glucocorticoid levels and scaled and centred glucocorticoid levels before analysis. We fit the model using the *brms* package in R [40], with a Gaussian link function, weakly informative prior slopes with mean 0 and standard deviation 1, 4 chains, and 10,000 iterations including 5,000 warmup iterations.

To test whether glucocorticoid levels were associated with daily habitat selection, we used integrated step selection analysis (iSSA; Avgar et al [41]). All habitat selection models, including iSSA, quantify the relative probability of selection for habitats using logistic regression, where the distribution of habitat values at used locations is compared to another sample of habitat values at available locations. Step selection analysis is a type of habitat selection analysis in which available locations are drawn from empirical distributions of step length and turn angles at each used location, thereby constraining available locations to the step level. In traditional step selection analyses, available steps are considered independent of habitat, while in iSSA they are sampled from pre-specified distributions of turn angles and step lengths parameterized on observed steps [41]. Constraining available steps in this way accounts for the fact that movement is also conditional on habitat selection [41]. This constraint also makes it possible to test the effect of temporally variable factors like glucocorticoid levels on habitat selection.

Our iSSA models tested for a relationship between glucocorticoids and habitat selection, whether the relationship between glucocorticoids and habitat selection changed after calving, and whether the relationship depended on days since calving. Our model included distance to forest and shrubland at the end of each step, an interaction between distance to forest and shrubland and FGMs at the start of the movement bout, a three-way interaction between the distance to cover-FGMs interaction and period before or after calving, and another three-way interaction between the distance to cover-FGMs interaction and time since calving.

Because we were interested in the effects of glucocorticoids on future habitat selection, we included only location points in our iSSA models that could have been influenced by measured FGMs. For most ungulate species including elk, circulating glucocorticoids are metabolized during approximately 20 h before defecation [42–44]. This means our FGM measurements represent the integrated peaks and troughs of circulating glucocorticoids over the 20 h before the elk deposited the sample. To limit our inference to only the effects of glucocorticoids on habitat selection, and not the effects of habitat selection on glucocorticoids, we subsampled GPS data to the 20–h preceding each sample (i.e., within the period when circulating glucocorticoids were being metabolized).

We sampled available steps from gamma distributions (turn angles) and von Mises distributions (step lengths) parameterized with movement characteristics of used steps [41]. We determined how many available steps were required to estimate selection coefficients by repeatedly fitting the model using ratios of between 1 and 1,000 available: used steps. Finally, to account for a possible correlation between samples from individuals [45], differences in sample size among individuals, and individual differences in habitat selection [46], we included random intercepts for movement bouts and random intercepts and slopes for all fixed effects and interactions. However, random effects models are challenging to fit within the conditional logistic regression framework typically used in step selection analysis because of the large number of step-specific strata. To deal with this challenge, we reformulated the conditional logistic model as a Poisson model with large, stratum-specific fixed intercepts as described in Muff et al. [47] using the *glmmTMB* package in R [48].

We used relative selection strength (RSS) as a measure of habitat selection effect size [49, 50]. We calculated RSS across the 0.2–0.8 quantile range of FGM levels in the population (approximately 1,200–2,600 µg•g^− 1^). RSS quantifies the ratio of the relative strength of selection for one location compared to selection at another location. When a single habitat characteristic varies between locations, RSS quantifies the change in selection for that characteristic [49]. In our case, we quantified the RSS for distance to cover habitat at the 0.2 quantile FGMs versus a range of FGM values over the 0.2–0.8 quantile range. The difference in selection strength across this range predicts the change in effect size for selecting distances further from forest and shrubland as FGMs increase. We compared the difference between these effect sizes by calving period while holding days since calving constant at zero, and differences by days since calving while holding calving period constant at post-calving.

We validated our iSSA model with used-habitat calibration (UHC) plots using the *uhcplots* package in R [51]. UHC plots measure model calibration, i.e., the agreement between distributions of habitat values at observed locations and distributions of habitat values at locations predicted as used by the model. UHC plots also compare used distributions to the distributions of habitat values at available locations to determine whether model covariates are important for predicting selection. Unlike other methods, UHC is appropriate for validating stratified habitat selection analyses like iSSA [51].

Our results are contingent on the assumption that we correctly identified fecal pellet samples belonging to individual elk and whether we knew whether samples came from before or after calving. To ensure our results were robust to potential fecal pellet misidentifications, we fit an additional iSSA and Bayesian generalized linear model, using the covariates in our original models, but including only samples we could attribute to individuals using DNA. To account for potential calving event misclassifications that might have biased our model estimates, we also fit an additional iSSA model including only samples collected at least 5 d before or after estimated calving events, i.e., those outside the window of our estimated calving events.

## Results

Our machine learning approach identified a calving period for 10 unconfirmed calving events. All five elk with unconfirmed events in 2019 were also confirmed pregnant by serum progesterone levels ≥ 3.7 ng•ml^− 1^ in blood samples collected at the time of capture (range 3.76–7.46 ng•ml^− 1^; Willard et al [52], supporting predictions from our machine learning models. The calving dates we retained for having exceeded our threshold for a positively identified calving period fell within a mean range of 5.0 days (± SE 1.1). The mean and SE of predicted dates for individual unconfirmed calving events are available in Table S1.

We included a final 76 fecal glucocorticoid metabolite (FGM) samples in our analyses from between May 14 and August 16 in 2019 and 2020, 32 of which were positively identified using DNA and 44 using machine learning. We included FGM samples collected up to 28 days before calving and up to 84 days after. The 122 samples we could not identify with DNA needed to pass several screening criteria before inclusion. First, we discarded 85 samples because they did not meet our 77% machine learning accuracy threshold. Of these discarded samples, we were able to recover 7 that met our 77% threshold for belonging to a different collared individual, bringing our total number of discarded samples down to 78 of 122. We combined the acceptable 44 machine learning-identified samples with the 32 DNA-identified samples for 76 samples in our Bayesian GLM models. Each of the 13 collared elk had anywhere from a single FGM sample, up to 16 FGM samples each (median = 4). For 13 calving events, individuals had samples only from either the pre- or post-parturition period (Figure S1).

We only included 68 of the final 76 samples in our iSSA models, 32 of which were identified using DNA, and 36 of which were identified using machine learning. We needed to exclude 8 of 76 FGM samples because they did not have enough associated location data (< 3 location points) to estimate a turning angle. The final 68 samples in our iSSA dataset were each associated with between 14 and 554 location points (median = 153). We used a ratio of 40 available: used points for all models as our sub-analysis suggested model coefficient estimates and standard errors remained relatively consistent from 30 to 100 available: used points (Figure S2). Though individual sample sizes and location points per individual were few, small samples are still sufficient for RSF inference when selection strength is strong and landscape heterogeneity is low (Street et al. 2021).

**Fig. 1:**
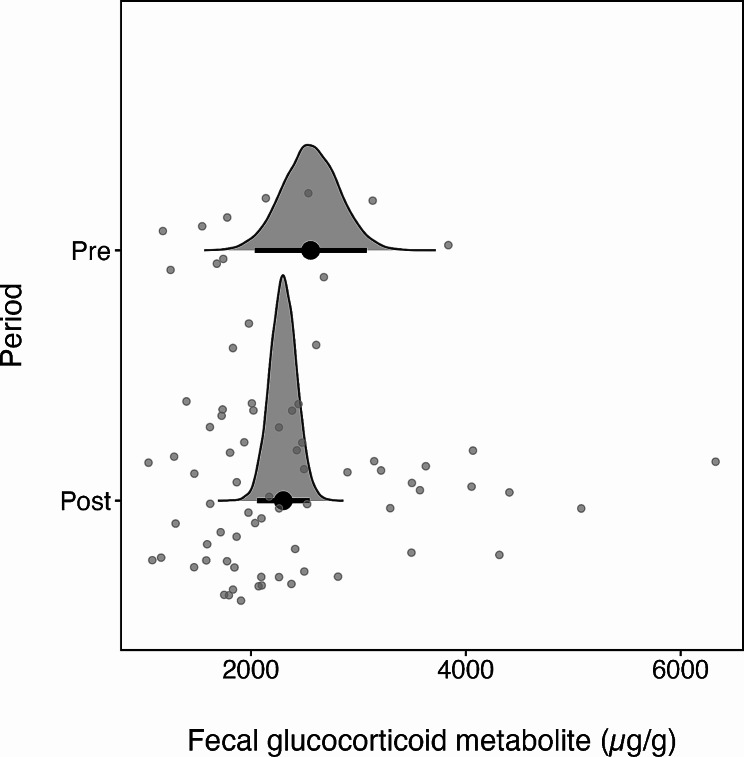
Half-eye plots comparing the distributions of glucocorticoid metabolite concentrations in fecal samples before (pre) and after (post) calving. Grey points are the glucocorticoid metabolite concentrations in each sample (*n* = 68), black points are the medians of the posterior distributions, and black intervals are the 95% quantile intervals of the posterior distributions

**Fig. 2:**
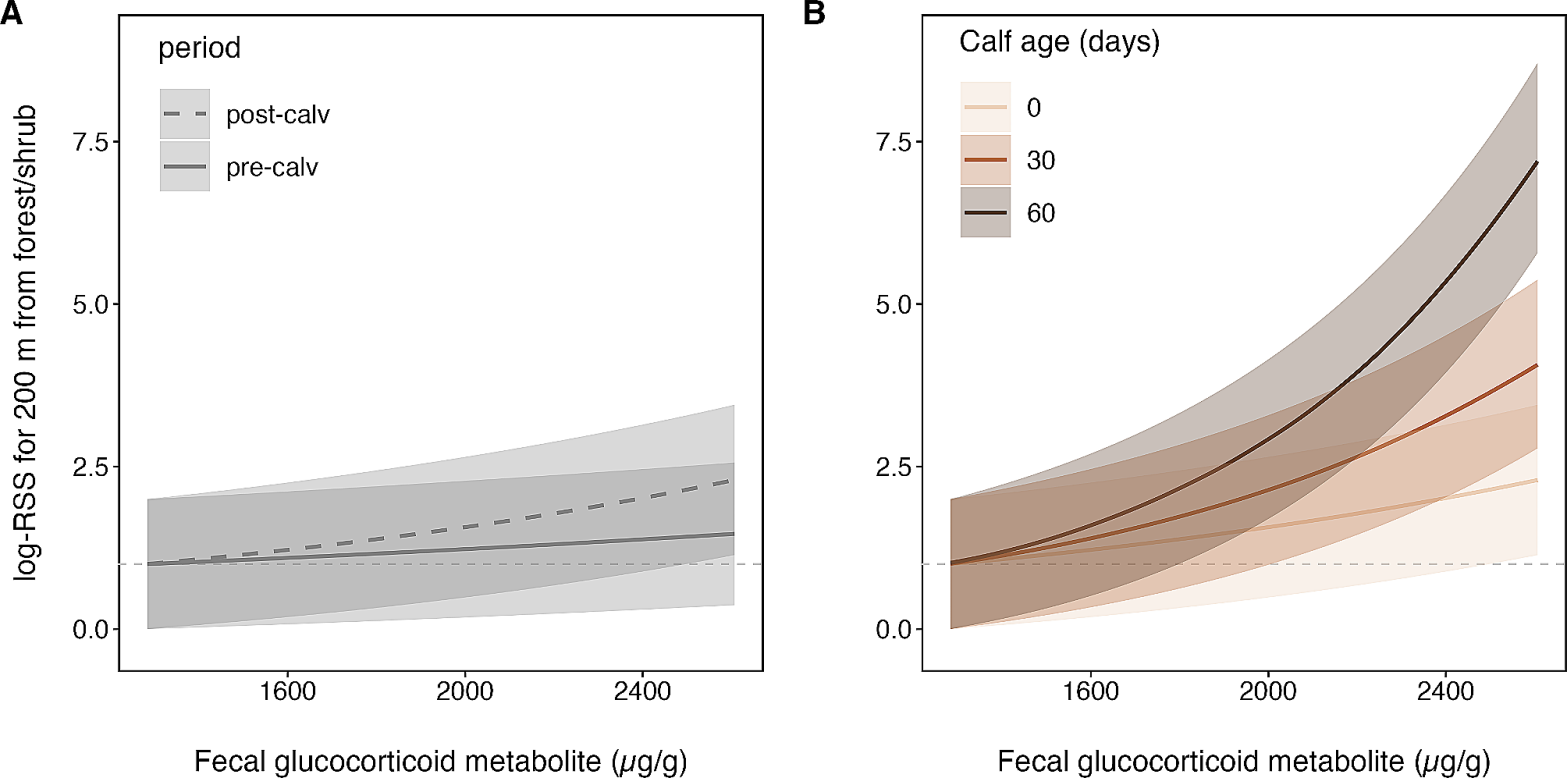
Log relative selection strength (RSS) for a location within forest and shrubland versus a location 200 m from cover with increasing glucocorticoid levels. In general, higher glucocorticoid levels predict greater selection for locations further from forest and shrubland, and thus closer to cropland. Panel A compares the RSS for locations relative to forest and shrubland before calving (pre-calv) and after calving (post-calv). Panel B compares RSS for these locations on the day of calving with calves aged 30 and 60 days. Solid lines are the mean predicted RSS and ribbons are 95% bootstrapped confidence intervals

We found no difference in glucocorticoid levels before and after calving (*n* = 76, estimate = 0.26, 95% CrI − 0.32, 0.85), though variation in glucocorticoids was higher after calving (Fig. 1). We also found a weak effect of year on glucocorticoids, which was larger in 2020 than 2019 (*n* = 76, estimate = 0.39, 95% CrI − 0.06, 0.83). The effect of year was no longer significant in our model including only DNA-identified samples, but this model similarly found no difference in glucocorticoid levels before and after calving (Table S2). Despite no overall difference in production before and after calving, selection for locations relative to forest and shrubland depended on glucocorticoid levels and changed over the calving season. In general, elk selected for locations closer to shrubland and forest (*n* = 68, e^ß^ = 0.90, 95% CI 0.82, 0.99). They were 50% more likely to select locations further from shrubland and forest, and thus further into cropland, for each unit increase in glucocorticoid levels (*n* = 68, e^ß^ = 1.44 95% CI 1.01, 2.04). This selection for cropland did not change immediately after calving when nutrition requirements were high (*n* = 68, e^ß^ = 0.80, 95% CI 0.50, 1.28; Fig. 2a). However, elk exhibited gradually stronger selection for locations further from forest and shrubland with days after calving (*n* = 68, e^ß^ = 1.01, 95% CI 1.00, 1.02; Fig. 2b).

Our UHC model validation supported our iSSA model inferences, as model coefficients discriminated used from available locations. The model was well calibrated, with observed habitat use close to that predicted by models, and differences in the distribution of used and available locations with distance to shrubland and forest (Figure S3). Our inferences were also robust to potential fecal sample misidentifications and calving date misclassifications; the directions and approximate magnitudes of effects from iSSA models did not change when we included only DNA-identified samples nor when we excluded samples collected within 5 d of estimated calving dates (Table S3).

## Discussion

We used habitat selection models to test for a relationship between glucocorticoids and changing selection for foraging habitats by female elk. Glucocorticoids encourage foraging by stimulating hunger in response to daily and seasonal changes in energy requirements [6, 10]. As predicted, we found elk with elevated daily glucocorticoids selected more for cropland, the habitat in our study system with the densest forage. We did not find that glucocorticoids increased after calving when seasonal energy requirements were greatest (Fig. 1), nor did we find an immediate change in the relationship between daily glucocorticoids and habitat selection after calving (Fig. 2a). However, the positive relationship we found between glucocorticoids and cropland selection did become stronger with time since calving (Fig. 2b), suggesting glucocorticoids might exert seasonally stronger effects on foraging commensurate with the energetic needs of growing calves. Together, our results suggest that changing glucocorticoid levels in our elk population reflect daily and seasonal changes in energy requirements.

We found elevated glucocorticoids were associated with stronger selection for cropland, the habitat in our study system that provides the most forage for elk. The association we detected between glucocorticoids and cropland selection is consistent with the role of glucocorticoids in regulating hunger and foraging behaviour [6,7]; elk with elevated glucocorticoids presumably required more energy and responded by selecting habitat that could fulfill those energy requirements. Others have found negative coarse-scale spatial associations between high-quality forage and glucocorticoid levels [53, 54]. These negative spatial associations between high-quality forage and glucocorticoids demonstrate an expected decline in glucocorticoids once energy requirements have been fulfilled; glucocorticoid levels drop following feeding and when energy stores are large [55]. This interpretation aligns with our findings. However, unlike many studies we connected glucocorticoid samples to individual animals, allowing us to control for variation in glucocorticoid-habitat relationships caused by individual differences in glucocorticoid production. We also considered only glucocorticoids from before observed habitat selection, bringing us closer to a causal effect of glucocorticoids on habitat selection. We therefore provide the best evidence to date that energy requirements influence glucocorticoid production, hunger, feeding behaviour, and habitat selection under natural conditions.

We predicted either an abrupt shift in glucocorticoid levels after calving or an immediately stronger association between foraging habitat selection and glucocorticoids, reflecting the immediate increase in energy required for lactation in elk [23]. Glucocorticoid changes associated with seasonal energy requirements are typical in many species [10], with many birds, for example, exhibiting stark changes in glucocorticoids upon egg-laying and up to offspring independence [56]. We may not have detected abrupt changes in glucocorticoids or habitat selection in our study because mammals exhibit different patterns of glucocorticoid production from non-mammals during gestation. In pregnant mammals, glucocorticoid levels tend to increase gradually toward parturition to facilitate fetal development [57]. The gradual increase in glucocorticoids in the pre-calving period could have prevented us from detecting any abrupt difference in glucocorticoids before and after calving. Circulating CBG levels also rise in concert with glucocorticoids in the latter part of pregnancy [58], which could have suppressed any behavioural changes associated with elevated glucocorticoids immediately after calving.

Despite no immediate changes in glucocorticoids or habitat selection after calving, we did find the relationship between glucocorticoids and foraging habitat selection became stronger for elk with older calves. When glucocorticoid levels were elevated, selection for cropland by elk with 60-day-old calves was nearly three-fold stronger than elk with newborn calves (Fig. 2). Elk calves double in weight within their first 50 days [23], so the stronger relationship between cropland selection and glucocorticoids seems to be related to calf energy demands. It is possible the relationship became stronger after parturition because glucocorticoids remained elevated after parturition, an effect that can persist relative to non-pregnant individuals for months [58]. It is also possible that a drop in CBG concentration after calving contributed to the stronger relationship between glucocorticoids and habitat selection.

We were not able to establish whether glucocorticoids cause elk with older offspring to forage more in cropland. However, the relationship between glucocorticoids and habitat selection suggests further investigation into a causal relationship is warranted. We suggest future studies integrate glucocorticoids into habitat selection models using experimental approaches, such as observing habitat selection after injection with synthetic glucocorticoids. Such approaches might help confirm a causal relationship between glucocorticoids and habitat selection when energy requirements increase.

## Conclusions

Glucocorticoids are often labelled stress hormones, produced in large amounts only by animals inhabiting stressful environments. However, our present understanding of glucocorticoid physiology suggests glucocorticoids are only peripherally related to stress [32] and instead regulate important responses to daily and seasonal energy requirements [8, 10]. In our study, we demonstrated a relationship between energy requirements and glucocorticoid levels in elk by finding individuals with elevated daily glucocorticoids selected more for foraging habitat. This relationship became gradually stronger in response to the seasonal energy demands of lactation. While we could not determine a causal effect of glucocorticoids on foraging habitat selection, our use of mechanistic habitat selection models and pairing glucocorticoid samples with individuals before observing their behaviour brought us closer to causal inference than typical correlative studies. Integrating glucocorticoids into mechanistic habitat selection models is a first step toward true causal inference and a better understanding of the fitness implications of elevated glucocorticoid levels under natural conditions.

### Electronic supplementary material

Below is the link to the electronic supplementary material.

Supplementary Material 1

## Data Availability

Movement data are not publicly available due to a government embargo but can be made available upon reasonable request from the Movebank data repository (Movebank ID 1265606810). All data and code required to replicate the analyses in the manuscript are publicly available at 10.5281/zenodo.8353419.
